# Body modifications in borderline personality disorder patients: prevalence rates, link with non-suicidal self-injury, and related psychopathology

**DOI:** 10.1186/s40479-023-00213-4

**Published:** 2023-03-02

**Authors:** Martin Blay, Roland Hasler, Rosetta Nicastro, Eléonore Pham, Sébastien Weibel, Martin Debbané, Nader Perroud

**Affiliations:** 1 ADDIPSY, Outpatient Addictology and Psychiatry Center, Santé Basque Développement Group, Lyon, France; 2 Claude Bernard University Lyon 1, Lyon, France; 3 Service of Psychiatric Specialties, Department of Mental Health and Psychiatry, University Hospitals of Geneva, Geneva, Switzerland; 4 Department of Psychiatry, University Hospitals of Strasbourg, Strasbourg, France; 5 Inserm u1114, Strasbourg, France; 6 Developmental Clinical Psychology Research Unit, Faculty of Psychology and Educational Sciences, University of Geneva, Geneva, Switzerland; 7 Research Department of Clinical, Educational and Health Psychology, University College London, London, UK; 8 Department of Psychiatry, University of Geneva, Geneva, Switzerland

**Keywords:** Borderline personality disorder, BPD, Body modification, Tattoo, Piercing, Non-suicidal self-injury, NSSI

## Abstract

**Background:**

Borderline Personality Disorder (BPD) is a potentially severe personality disorder, characterized by difficulties in emotion regulation and control of behaviors. It is often associated with non-suicidal self-injury (NSSI). Borderline personality features have also been linked to body modifications (BMs). However, the prevalence of BMs, the link between BMs and NSSI, and between BMs and several psychopathology dimensions (e.g. borderline severity, emotion regulation, impulsivity …) remains understudied in patients with BPD. This study aims to fill this gap, and to provide further evidence on the link between NSSI and BMs.

**Methods:**

We used data from a psychiatric outpatient center located in Switzerland (*n* = 116), specialized in the assessment and treatment of BPD patients. Patients underwent several semi-structured interviews and self-report psychometric scales at the arrival, and the data were retrospectively analyzed.

**Results:**

We found that 70.69% of the patients had one piercing or more, and 69.83% were tattooed. The total score of body modifications and the total number of piercings score of piercings were significantly positively associated with NSSI and the SCID BPD total score. The association with the SCID score was mainly driven by the “suicide and self-damaging behaviors” item and the “chronic feeling of emptiness” item. A significant association was found between total number of piercings and emotion dysregulation. On the other hand, the self-reported percentage of body covered by tattoos score was specifically associated with the sensation seeking subscale of the UPPS-P.

**Conclusion:**

This study provides evidence on the prevalence of BMs in BPD patients, and on the link between BMs and NSSI in this population, suggesting a role of emotion regulation in the link between both constructs. These results also suggests that tattoos and piercings may be differentially linked to specific underlying psychological mechanisms. This calls for further considerations of body modifications in the assessment and care of BPD patients.

**Supplementary Information:**

The online version contains supplementary material available at 10.1186/s40479-023-00213-4.

## Background

Borderline personality disorder (BPD) is a common personality disorder, affecting around 1% of the general population, around 10% of outpatient psychiatric population, and around 20% of inpatient psychiatric population [1]. It is associated with low functional, social and professional outcomes, resulting in an overall decreased quality of life [2]. In the DSM-5, BPD is defined as “a pervasive pattern of instability of interpersonal relationships, self-image, and affects, and marked impulsivity beginning by early adulthood and present in a variety of contexts” [3]. As this definition highlights, the major symptomatic dimensions of BPD are emotion regulation, impulsivity, identity disturbance, and interpersonal issues. Also, BPD patients often present non-suicidal self-injury (NSSI), which can be defined as the deliberate, self-inflicted destruction of body tissue without suicidal intent and for purposes not socially sanctioned, and includes behaviors such as cutting, burning, biting and scratching skin [4]. NSSI behaviors can occur in the general population [5] but have a higher prevalence in BPD patients [6] in relation to BPD-relevant symptomatic dimensions such as impulsivity [7] or emotion regulation [8].

Body modifications (BMs) can be defined as the purposeful alteration of the body, often for artistic or aesthetic reasons [9]. The two forms of body modification considered in this work are tattooing and piercing. Although these practices have existed for thousands of years, tattooing and piercing have more often been negatively appraised within Western Society until recent decades, where their prevalence has increased and stigma reduced [10, 11]. Studies find that the prevalence of body piercing can reach 51% in student samples [12], whereas prevalence of tattoos was estimated to 18.5% in a recent international study evaluating the prevalence of tattooing among a representative sample of the population of 5 major countries [13]. More recently the potential associations between body modifications and psychopathology have been examined, with many studies finding a link between BMs and a wide range of psychopathological dimensions (e.g. self-esteem [14], impulsivity [15], substance abuse [16], suicide [17]…), whilst other studies do not find any relationship [18]. In fact, some authors consider that the psychopathological significance of BMs depends on the patient’s profile and history, and ranges from no signification to potentially informative of the underlying psychopathology [19]. Also, another question of interest when considering BMs and psychopathology is the nature of the relationship between NSSI and BMs [20]. Despite the exclusion of BMs from the NSSI definition by the International Society for the Study of Self-Injury (which states that “behaviors such as body modification, body piercing, tattooing, and religious self-flagellation are not usually considered forms of self-injury” [21]), other authors consider that tattooing and piercing can be considered as socially accepted forms of NSSI [22, 23]. Indeed, many studies have suggested that BMs can be used as a mean of emotion regulation (*e.g.* [19, 20, 24, 25]. For example, Stirn & Hinz (2008) formulated the hypothesis that, in their groups of volunteers wearing BMs, among those who reported having engaged in NSSI in their childhood (27% in their sample), some could use body modifications as a substitute for their auto-aggressive behaviors, and some former self-cutters even attributed “therapeutic significance” to their BMs.

Considering body modifications in BPD patients, to our knowledge, no study has specifically examined the prevalence rates of body modifications in borderline patients. Concerning the link between BPD and body modifications, the few available studies on the relationship between both found a significant association between BPD pathology or features and engagement in body modifications [26, 27]. More precisely, in a student sample, D’Ambrosio and colleagues found higher Borderline Syndrome Index (BSI) mean values in subjects with piercings and tattoos compared to patients without body modifications. Also, in a community sample, Vizgaitis & Lenzenweger found a positive correlation between BPD features (measured by the International Personality Disorder Examination—Screen, IPDE-S) and the total number of body modifications, piercings, tattoos and scarifications, with a partial mediation of identity diffusion and low self-concept clarity in this relationship. Finally, the current literature also lacks investigations examining the psychopathological specificities of BPD patients wearing BMs. Yet, this kind of research could be useful for a better clinical understanding of the experience of BPD patients wearing BMs.

Thus, the current study aims to contribute to fill these gaps, by exploring body modifications in BPD patients, and especially the link with NSSI. The primary objective was to provide insights regarding the prevalence rates of body modifications in BPD patients and to compare it with a clinical control group. The secondary objective was to study the link between BMs and NSSI among patients with BPD and to provide specific elements on the potential common nature between the two. The third objective was to study the link between BMs and several clinical dimensions (e.g. borderline symptomatology severity, depression, anxiety, emotion regulation, impulsivity…) in order to provide insights regarding clinical dimensions in this particular population.

## Methods

### Population

Data were collected between 2018 and 2021 in an outpatient unit specialized in the assessment and treatment of borderline personality disorder (BPD) and adult attention deficit hyperactivity disorder (ADHD), the TRE Unit, in the University Hospitals of Geneva, in Switzerland. Patients are usually referred by their general practitioner, psychiatrist, psychologist, or other mental health care professional for one or both disorders.

The inclusion criteria for participation in the present study were: 1°) Being referred to the unit for assessment and/or care of adult ADHD, BPD, or emotion dysregulation 2°) Being at least 18 years old 3°) Having a diagnosis of BPD made with the SCID-5-PD and 4°) Providing informed consent for participation in the study and use of health data for research purposes. Moreover, participants at least 18 years old without a diagnosis of BPD but with a diagnosis of adult ADHD made with the ACE + were also included in the present study to serve as a clinical control group regarding prevalence of body modifications. The study was approved by the Ethics Committee of the Geneva University Hospitals.

### Procedure

Patients were assessed for BPD using the Structured Clinical Interview for DSM-V Personality Disorders (SCID-5-PD, [28]). The SCID-5-PD was administered by trained psychologists. Each criterium was noted as present or absent, and if a minimum of five out of nine criteria was met, the BPD diagnosis was made. Control were assessed for adult ADHD using the ADHD Child Evaluation for Adults (ACE + , [29]). The ACE + was also administered by trained psychologists. Each criterium was noted as present or absent, and the diagnosis of ADHD was made if the score on either attention or hyperactive/impulsive symptoms were at least of 5/9. Other psychiatric disorders were clinically assessed, including major depressive disorder, bipolar disorder, anxiety disorder, obsessive–compulsive disorder, post-traumatic stress disorder, autism spectrum disorder, and substance use disorder. Medical records and information provided by other clinicians involved in patients’ care were reviewed to assess the lifetime comorbidities.

Assessment of NSSI behaviors, body modifications and suicidal behaviors**.** We designed a new scale for this study, the Suicidal Behavior and Body Damage & Modifications Scale (SBBDM-S), to assess the *lifetime* presence of NSSI and BMs. The SBBDM-S is a 10-items self-report questionnaire used to investigate the lifetime prevalence and the past number of suicidal behaviors, body modifications (including tattoos and piercing) and/or NSSI. The translated questionnaire is available in the [Additional File 1]. The suicidal behaviors are studied in question 1 to 4 (with the question 1 and 2 assessing respectively the lifetime presence and the number of suicide attempts, question 3 assessing the age when the first suicide attempt occurred, and question 4 assessing the method used in the most serious attempt). Body modifications are studied in questions 5 to 8 (with question 5 and 7 assessing respectively the lifetime acquisition of a tattoo and of a piercing by a professional (including earlobe piercing), the question 6 assessing the tattoo-covered body surface percentage (< 10%, 10–50%, 50–90%, > 90%), and the question 8 assessing the total number of piercings). The NSSI were assessed in question 9 and 10 (question 9 assessing the lifetime presence of NSSI, and question 10 assessing the number of times each type of NSSI type occurred). The total number of NSSI was calculated by summing all the individual numbers of times each type of NSSI occurred.

For the purposes of the analyses, we defined three sub-scores: the total number of piercings, the tattoo-covered body surface percentage, and the total score of body modifications. For the rest of the article, these three subscores will be named as PercTot, TatTot and BMTot, respectively. These three subscores were treated as ordinal scales. For PercTot, the original scale had the following characteristics: mean = 2.89, SD = 3.44 and median [range] = 1 [0–15]. Given the low number of patients in the higher categories, the scale was treated as follows in order to balance the number of patients across groups: 0 = no piercing (*N* = 34), 1 = one piercing (*N* = 21), 2 = two piercings (*N* = 16), 3 = three or more piercings (*N* = 45). For TatTot, the original scale had the following characteristics: mean = 0.95, SD = 0.79 and median [range] = 1 [0–3]. Once again, the scale was reduced to a three-point ordinal scale, and the tattoo-covered body surface percentage of > 10%, 50–90% and > 90% were put together as follows: 0 = no tattoo(*N* = 35), 1 = less than 10% of the body covered (*N* = 55), 2 = more than 10% of the body covered (*N* = 26). Finally, for BMTot, corresponding to the sum of the *original* PercTot & TatTot scores, the original scale had the following characteristics: mean = 3.84, SD = 3.69 and median = 2 [0–17]. Only 25 subjects had a score above six and were thus regrouped with those having a score of 6 on a seven-point ordinal scale, from 0 (*N* = 17) to >  = 6 (*N* = 27). Finally, the second item of the SBBDM-S was used to assess the total number of suicide attempts.

### Assessment of psychopathological domains

For BPD severity, the French version of the Borderline Symptom List – 23 items (BSL-23) was used [30, 31] The BSL-23 questionnaire is a self-report questionnaire used to investigate the global severity of BPD symptoms, and it is also used to estimate the effect of therapy in BPD patients. It consists in 23 items investigating the *last week’s* symptomatic experience of BPD patients, with a 5-point Likert response format (from 0 (“never”) to 4 (“always”)), and the mean score is calculated. A classification of BPD severity was recently proposed based on the mean score at the BSL-23 [32].

For emotion regulation, the French versions of the Difficulties in Emotion Regulation Scale – 18 items (DERS-18; [33]) and of the Cognitive Emotion Regulation Questionnaire (CERQ, [34, 35]) were used. The DERS-18 is a self-report questionnaire, the short form created in 2016 of the original DERS, and assesses *in the last month* the intensity of different domains of emotion regulation: lack of awareness of one’s emotions (awareness), lack of clarity about the nature of one’s emotions (clarity), lack of ability to engage in goal-directed activities during negative emotions (goals), lack of ability to manage one’s impulses during negative emotions (impulse), lack of acceptance of one’s emotions (nonacceptance) and lack of access to effective emotion regulation strategies (strategies). It consists in 18 items relative to the presence of these domains, with a 5-point Likert response format (from 1 (“almost never”) to 5 (“almost always”)), and the sub-scores were made following the original article recommendations. Also, a total score can be calculated by summing all the sub-scores, with a higher score indicating greater difficulties with emotion regulation. For this study, we only considered the DERS total score. On the other side, the CERQ is a self-report questionnaire, assessing 9 different emotion regulation strategies: acceptance, positive refocusing, refocus on planning, positive reappraisal, putting into perspective, blame, rumination, catastrophizing and blaming others. It consists in 36 items relative to these domains, with a 5-point Likert response format (from 1 (“almost never”) to 5 (“almost always”)). Five of these strategies (acceptance, positive refocusing, refocus on planning, positive reappraisal, putting into perspective) can be summed in a CERQ adaptative sub-score, while the remaining four (blame, rumination, catastrophizing and blaming others) can be summed in a CERQ non-adaptative sub-score. For this study, we only considered the CERQ adaptative and non-adaptative sub-scores.

For impulsivity, the French version of the short form of the UPPS-P Impulsive Behavior Scale [36, 37] was used. The UPPS-P is a self-report questionnaire assessing five domains of impulsivity: negative urgency, positive urgency, lack of premeditation, lack of perseverance and sensation seeking. It consists in 20 items related to these domains, with a 4-point Likert response format (from 1 (“totally agree”) to 4 (“totally disagree”)), and the sub-scores were made following the original article recommendations.

For depressive symptoms, the French version of the Beck Depression Inventory was used (BDI, [38, 39]. The BDI is a self-report questionnaire assessing the intensity of depressive symptoms, and consists in 21 items, with a 4-point Likert response format.

For anxiety, the French version of the State Trait Anxiety Inventory (STAI, [40] was used. The STAI is a 40 items self-report questionnaire assessing the intensity of anxiety as a trait (i.e. *generally*, 20 items with a 4-point Likert response format) and as a state (i.e. *these days,* 20 items with a 4-point Likert response format). For this study, we only considered the STAI-trait score.

### Statistical analyses

We used ordinal logistic regression to test the associations between body modification variables (PercTot, TatTot, BMtot) treated as ordinal scales and clinical variables (history of suicide attempts, NSSI, SCID total score, BSL-23, BDI, STAI, CERQ adaptive and non-adaptive subscales, DERS total score, UPPS-P subscales). We adjusted our models on age and gender when required. As we conducted several tests (one for each non-correlated variable, with the CERQ subscales, the UPPS-P subscales, and the three BMs variables being correlated variables, *n* = 10) for the same dependent variables, statistical significance was accepted for *p* values < 0.05/10 = 0.005. We also examined the correlation between variables using Spearman's correlations. Finally, we used Chi-square tests to compare percentage between groups and Student t-tests to compare mean scores between groups Analyses were conducted with StataSE 16.0 [41].

## Results

### Descriptive characteristics of the sample

The clinical and demographic characteristics can be found in Table 1. A total of 116 patients met the criteria for enrollment in the study. The mean age was 29.64 years (SD = 9.59). Most of the patients were females (78.45%), single or living alone (56.89%), without children (73.27%), and had professional activity (63.79%). The main lifetime comorbidities were substance use disorder (61.21%), major depressive disorder (54.31%), anxiety disorder (53.44%), and adult attention deficit hyperactive disorder (46.55%). 61.21% of our sample reported a previous suicide attempt. Finally, concerning NSSI, the mean number was of 13.18 (SD = 9.48) with most of the patients reporting at least one NSSI in their life (*N* = 101; 87.07%). To note, the ADHD patients we used as a control group to allow comparisons on body modifications prevalence were mostly male (*n* = 68, 56.67), with a mean age was of 35.54 (SD = 11.86).

**Table 1:** Clinical and demographic characteristics of the 116 BPD patients

Variables	N (%)
**Gender (female)**	*91 (78.45%)*
**Marital status (single or living alone)**	66 (56.89%)
**Children (without)**	85 (73.27%)
**Professional activity (without)**	42 (36.21%)
**Lifetime comorbidities**	
Major depressive disorder	63 (54.31%)
Bipolar disorders	9 (7.76%)
Anxiety disorder	62 (53.44%)
Substance use disorders	71 (61.21%)
ADHD	54 (46.55%)
Eating disorders	38 (32.75%)
**SBBDM-S**	
History of suicide attempt	71 (61.21%)
History of NSSI	101 (87.07%)
**Scale**	**Mean score (SD)**^a^
**SBBDMS-S**	
Total number of NSSI	13.18 (9.48)
**SCID-BDL**	6.85 (1.42)
**BSL-23**	2.04 (0.89)
**BDI**	33.21 (11.73)
**STAI—trait**	62.88 (8.50)
**DERS – 18 Total score**	63.05 (17.45)
**CERQ**	
Adaptive cognitive regulation strategies	51.92 (13.62)
Non-adaptive cognitive regulation strategies	50.40 (9.73)
**UPPS-P**	
Negative Urgency	12.84 (2.85)
Positive Urgency	13.08 (2.65)
Lack of Premeditation	9.69 (3.22)
Lack of Perseverance	10.02 (3.21)
Sensation-Seeking	11.34 (3.41)

*Abbreviations*: *ADHD *Attention deficit hyperactive disorder, *BDI*  Beck depression inventory, *BSL-23 *Borderline symptom list – 23 items, *CERQ *Cognitive emotion regulation questionnaire, D*ERS-18 *Difficulties in emotion regulation scale – 18 items, *NSSI *Non-suicidal self-injury, *SBBDM-S *Suicide behaviors and body damage & modifications scale, *SCID-BDL *Structured clinical interview for DSM-V personality disorders, *STAI *State Trait Anxiety Inventory, *UPPS-P *UPPS-P Impulsive behavior scale

^a^*Score are given two decimal places*

Concerning the psychometric scores, our population had a mean number of BPD DSM-5 criteria assessed by the SCID of 6.85 (SD = 1.42), with a mean BSL-23 score of 2.04 (SD = 0.89), indicating a high severity according to Kleindienst and colleagues [32]. The mean BDI score was 33.21 (SD = 11.73), indicating severe depressive symptomatology, and the mean STAI-trait score was 62.88 (SD = 8.50), indicating a high level of anxiety. The mean DERS-18 total score was 63.05 (SD = 17.45), and the mean CERQ-adaptative and non-adaptative sub-scores were, respectively, 51.92 (SD = 13.62) and 50.40 (SD = 9.73). Finally, for the UPPS-20, the negative urgency, positive urgency, lack of premeditation, lack of perseverance and sensation-seeking sub-scores were respectively 12.84 (SD = 2.85), 13.08 (SD = 2.65), 9.69 (SD = 3.22), 10.02 (SD = 3.21) and 11.34 (SD = 3.41).

As indicated in Table 2, for BPD patients, regarding piercings, 45 patients had three or more piercings (38.79%), 34 (29.31%) had none, 21 (18.10%) had one, and 16 (13.79%) had two. Regarding tattoos, 55 patients had less than 10% of their body covered by tattoos (47.41%), 35 had no tattoos (30.17%), and 26 had more than 10% of their body covered by tattoos (22.41%). Regarding the BMtot score, 23.28% of the patients had a score of 6 or more (the most common score in our sample) and 14.66% had a score of zero. Finally, when comparing to our ADHD control group, we found a significant and large difference between groups, with a global higher number of piercings, tattoos, and total number of body modifications in BPD patients.

**Table 2:** PercTot, TatTot and BMTot scores

Body modifications	BPD group (N, %) *n* = 116	ADHD control group (N, %) *n* = 120	Test value	*P* value
**PercTot score**			Chi2 = 25.371	** < 0.000**
0	34 (29.31%)	73 (60.83%)		
1	21 (18.10%)	16 (13.33%)		
2	16 (13.79%)	11 (9.17%)		
3	45 (38.79%)	20 (16.67%)		
**TatTot score**			Chi 2 = 33.990	** < 0.000**
0	35 (30.17%)	80 (66.67%)		
1	55 (47.41%)	33 (27.50%)		
2	26 (22.41%)	7 (5.83%)		
**BMTot score**			Chi2 = 41.466	** < 0.000**
0	17 (14.66%)	62 (51.67%)		
1	19 (16.38%)	16 (13.33%)		
2	18 (15.51%)	15 (12.50%)		
3	13 (11.21%)	9 (7.50%)		
4	11 (9.48%	3 (2.50%)		
5	11 (9.48%)	5 (4.17%)		
> = 6	27 (23.28%)	10 (8.33%)		

Associations with the total number of body modifications score (BMTot score) We found a significant association between age and BMTot (b=-0.54; *p*=0.001; 95%CI from -0.87 to -0.21), with younger subjects having a higher score on the total number of body modifications score. Also, there was a significant association between BMTot score and NSSI (b=0.73; *p*<0.0001;95% to 0.34 to 1.11). Moreover, we found a significant association between the BMTot score and the SCID BPD total score (b=0.52; *p*=0.002; 95%CI 0.20 to 0.85), mainly driven by item 5 “suicide and self-damaging behaviors” (b=0.89; *p*=0.012; 95%CI from 0.19 to 1.58) and item 7 “chronic feeling of emptiness” (b=0.81; *p*=0.053; 95%CI from -0.01 to 1.64). We also found a trend of association between BMtot and DERS total score (b=0.49; *p*=0.008; 95%CI from 0.12 to 0.84). All the other associations were non-significant (namely, between BMTot score and BSL-23 score, history of suicide attempts, BDI score, STAI-trait score, CERQ sub-scores or UPPS sub-scores; see [Additional File 2])

### Associations with the total number of piercings score (PercTot score)

Again, we found an association between age and PercTot (b = -0.57; *p* = 0.002; 95%CI from -0.92 to -0.21), with younger subjects having more piercings. Also, there was a significant association between PercTot score and NSSI (b = 0.69; *p* = 0.001; 95%CI from 0.28 to 1.09). Moreover, we found a significant association between PercTot score and the SCID BPD total score (b = 0.55; *p* = 0.002; 95%CI from 0.21 to 0.90*)* which was once again mainly explained by item 5 (b = 1.09; *p* = 0.003; 95%CI from 0.36 to 1.82) and item 7 (b = 0.88; *p* = 0.043; 95%CI from 0.03 to 1.74) of the SCID. We also found a significant association between PercTot score and the DERS total score (b = 0.81; *p* = 0.004; 95% from 0.26 to 1.35). Finally, we found a significant correlation between TatTot score and PercTot score (*r *= 0.51; *p* < 0.001)**.** All the other associations were non-significant (namely, between PercTot score and BSL-23 score, history of suicide attempts, BDI score, STAI-trait score, CERQ sub-scores or UPPS sub-scores; see [Additional File 2]).

### Associations with the tattoo-covered body surface percentage score (TatTot score)

We found a significant association with the subscale sensation seeking of the UPPS (b = 0.54; *p* = 0.004; 95%CI from 0.17 to 0.90). Also, we found a trend of association with the DERS total score, although not significant (b = 0.60; *p* = 0.012; 95%CI from 0.13 to 1.07) and other trends were observed for some of the other subscales UPPS: positive urgency (b = 0.36; *p* = 0.038; 95% from 0.02 to 0.71) and lack of premeditation (b = 0.42; *p* = 0.02; 95%CI from 0.07 to 0.78). All the other associations were non-significant (namely, between TatTot score and age, NSSI, SCID BPD score, BSL-23 score, history of suicide attempts, BDI score, STAI-trait score, CERQ sub-scores or “lack of perseverance” and “negative urgency” UPPS sub-scores; see [Additional File 2]).

### Subgroup analyses

Finally, we conducted subgroup analyses regarding clinical characteristics on two sub-groups of patients: those without body modifications (either tattoo or piercing, *n* = 17) and those with the highest number of body modifications (3 or more piercing and more than 10% of tattoo-covered body surface percentage, *n* = 14). Compared to the no BM group, we found a significant higher mean total number of NSSI (no BM = 6.94 (7.97); highest BM = 18.14 (9.13), *p* = 0.001), a significant higher mean number of SCID-BDL criteria (no BM = 6.24 (1.09); highest BM = 7.36 (1.39); *p* = 0.018), a significant higher DERS mean score (no BM = 55.37 (10.91); highest BM = 78.5 (38.83); *p* = 0.03), a significant higher mean positive urgency (no BM = 12.76 (2.05); highest BM = 14.40 (1.90); *p* = 0.03) and sensation-seeking (no BM = 9.96 (4.46); highest BM = 13.5 (2.88); *p* = 0.02) UPPS-P sub-scores. All the other results were non-significant. Detailed results can be found in Table 3

**Table 3:** Comparisons between patients without body modifications and patients with the highest level of body modifications. Patients

Variables	No BM (*n* = 17)	Highest number of BM (*n* = 14)	Test value	*P* value
**Gender (F, %)**	13 (76.47%)	12 (85.71%)	Chi2 = 0.42	0.517
**SBBDM-S**				
History of SA (n, %)	11 (64.71%)	11 (78.57%)	Chi2 = 0.71	0.397
History of NSSI (n, %)	11 (64.71%)	13 (92.86%)	Chi2 = 3.48	0.06
Total number of NSSI (mean, SD)	6.94 (7.97)	18.14 (9.13)	t =—3.65	**0.001**
**SCID BDL (mean, SD)**	6.24 (1.09)	7.36 (1.39)	t = -2.52	**0.018**
**BSL-23 (mean, SD)**	1.81 (0.64)	2.22 (1.13)	t = -1.26	0.219
**BDI (mean, SD)**	29.68 (11.04)	37.01 (12.70)	t = -1.72	0.09
**STAI-trait (mean, SD)**^a^	62.10 (8.19)	63.94 (9.67)	t = -0.56	0.58
**DERS-18 total score (mean, SD)**^**a**^	55.37 (10.91)	78.5 (38.83)	t = -2.28	**0.03**
**CERQ (mean, SD)**				
Adaptive cognitive regulation strategies	53.57 (17.34)	54.45 (14.63)	t = -0.15	0.88
Non-adaptive cognitive regulation strategies	48.45 (11.39)	51.67 (7.75)	t = -0.90	0.38
**UPPS-P (mean, SD)**				
Negative Urgency	12 (3.20)	13.36 (3.25)	t = -1.17	0.25
Positive Urgency	12.76 (2.05)	14.40 (1.90)	t = -2.29	**0.03**
Lack of Premeditation	9.59 (3.12)	10.71 (3.36)	t = -0.97	0.34
Lack of Perseverance^b^	10.5 (3.03)	11.08 (3.25)	t = -0.49	0.63
Sensation-Seeking	9.96 (4.46)	13.5 (2.88)	t = -2.56	**0.02**

*Abbreviations: BDI *Beck depression inventory, *BM* Body modifications, *BSL-23* Borderline symptom list – 23 items, *CERQ* Cognitive emotion regulation questionnaire, *DERS-18* Difficulties in emotion regulation scale – 18 items, *NSSI* Non-suicidal self-injury, *SBBDM-S* Suicide behaviors and body damage & modifications scale, *SCID-BDL* Structured clinical interview for DSM-V personality disorders, *STAI-trait* State trait anxiety inventory, trait subscale, *UPPS-P* UPPS-P impulsive behavior scale

^a^Data were missing in 1 patient in the no BM group

^b^Data were missing in 1 patient in the no BM group and 1 patient in the highest number of BM group in the highest number of BM group

## Discussion

This study aimed to provide insights in a BPD population into the prevalence of body modifications, the link between NSSI and body modifications, and into the potential clinical characteristics of the body-modified patient subtype. We found a high prevalence of body modifications in our sample, and several associations of interest. Notably, we found that the total score of body modifications (BMtot) and the total number of piercings scores (PercTot) (but not the tattoo-covered body surface percentage score, TatTot) were significantly associated with NSSI, and that these two scores also were significantly associated with the SCID total score (which was mainly explained by the items 5—“suicide and self-damaging behaviors”—and 7 – “chronic feeling of emptiness”). Also, we found that PercTot was significantly associated with the DERS total score, with a trend of association with the BMtot and with TatTot. Finally, we found a significant association between TatTot and the sensation-seeking subscale of the UPPS-P, with a trend of association with other UPPS subscales. Despite limitations, we believe that this work provides interesting results on body modifications in borderline patients, and we will discuss each outcome in detail in the following discussion.

In our study, 70.69% of the patients had at least one piercing, and 69.83% had at least one tattoo, with 22.41% having more than 10% of their body covered by tattoos. Several studies have assessed the prevalence of body modifications in the general population. In a 2002 study [12] evaluating the prevalence of body modifications among 481 students (with a mean age of 21), Mayers and colleagues found a prevalence of body piercing of 51%, and that of tattooing of 23%. In a more recent international study, Kluger et al. (2019) evaluated the prevalence of tattooing among a representative sample of the population of 5 major countries (*n* = 11 079) and found that, in the 25–34 years old subgroup (which can be compared in term of age to our sample), 27.3% of the sample had at least one tattoo (with 16.4% wearing one tattoo and 10.9% wearing several) which is far less than in our sample where 69.83% of BPD patients had at least one tattoo and 22.41% had more than 10% of their body covered. Concerning studies directly comparing BPD patients and the general population, only a few have been made, with conflicting results regarding this outcome (some finding a difference [42] and some not [43]). Given the lack of studies comparing body modifications in BPD patients with other clinical population, we also compared BM prevalence in BPD patients with BM prevalence in an adult ADHD clinical control group. We found a significant higher number of each type of body modifications *and* of total number of body modifications in BPD patients. Thus, even if we found a higher prevalence rate of body modifications in our BPD sample compared to published studies in the general population and compared to a clinical control group, these data suggest a lack of knowledge concerning the prevalence of body modifications in BPD patients, in itself and compared to the general and clinical population.

The positive association between BMtot and PercTot and NSSI suggest a close link between both in BPD patients. Concomitantly, we found a significant association between emotion dysregulation (as measured by the DERS total score) and the total number of piercings, with a trend of association for the total number of body modifications, suggesting that the more a patient is emotionally dysregulated, the more he will tend to have piercings or higher levels of body modifications. Moreover, when comparing patients without BM versus patients with the highest level of BM, we found a higher total number of NSSIs (around 2.6 times more) and a higher DERS mean score in the latter group. These results can be put in perspective with previous works. As presented in the introduction, one major hypothesis on the role of NSSI is as an emotion regulation mean [8], and BMs are considered by some authors as socially accepted forms of NSSI [23]. Thus, given the concomitant link we found between body modifications *and* NSSI or emotion regulation, one could infer that body modification can be used as an emotion regulation mean, like NSSI, which would be in line with previous works [19, 20, 24, 25].

Regarding impulsivity, in our overall sample, we did not find any positive association between our two BMs variables associated with NSSI (namely BMtot and PercTot) and any of the UPPS-P subscales, even though when we found higher positive urgency and sensation seeking mean scores in patients with the highest level of BM when comparing them with patients without BM. This absence of link can be surprising, given the fact that people wearing BMs have been shown to have a higher impulsivity in non-clinical population [15, 44], that people undergoing NSSI are shown to be more impulsive as well [7] and that BPD patients are also known to be more impulsive that the general population [45]. Thus, given these previous works, one could have inferred that BMs and NSSI may co-occur in BPD patients due to common predisposing factors, like impulsivity, and that the positive association we found can be -at least partly- explained by the higher impulsivity in BPD population*.* However, given the absence of association between our two BMs variables associated with NSSI (namely BMtot and PercTot) *and* any of the UPPS-P subscale, our results do not support this hypothesis.

It is also important to note that TatTot was not associated with NSSI or with BPD severity and/or symptoms but was significantly associated with sensation seeking subscales of the UPPS, with trends of association with the positive urgency and lack of premeditation subscales. First, when comparing the results concerning BMtot score and PercTot score, there seems to be different profiles of associations with the studied variables (with BMtot & PercTot being associated with NSSI and BPD symptoms while TatTot being associated with impulsivity). Second, we can also compare these results with previous studies that found a higher impulsivity in tattooed women compared to not-tattooed woman [15] and that sensation-seeking preference is predictive of number of tattoos but not number of piercings in college student population [44]. Thus, we can make the hypothesis that a small number of tattoos may not have clinical significance compared to a small number of piercings or a global higher number of body modifications in BPD patients. Finally, the positive association between TatTot and the sensation seeking sub-score *and* the trends of association with the positive urgency sub-score may partly explain the higher mean level of sensation seeking and positive urgency we found when comparing patients without BM and patients with the highest level of BM.

Taken together, these preliminary results suggest that BMs and NSSI may be linked, probably due to common factors like emotion regulation. However, further studies are needed to confirm/infirm this hypothesis and to precise the other potential factors involved.

Regarding the link with BPD symptoms and/or severity, we found positive associations between the PercTot and BMtot *and* the total number of BPD criteria. This result is in line with previous reports, that found that BPD features were positively correlated with body modifications [27]. It is also in line with the higher mean number of BPD criteria we found in the subgroup of patients with the highest levels of BM compared to the subgroup of patients without BM. However, contrary to our hypothesis, we did not find an association between body modifications, either cumulated in a total score or separated between tattoos and piercing, and the severity of BPD measured with the BSL-23. This difference of association can be linked to the fact that, contrary to the SCID (which explores the presence/absence of BPD symptoms), the BSL-23 measures the current severity of BPD and is more a scale measuring the current impact of the disease on the general psychological functioning of the patient than a mean of assessment of symptoms of BPD. But these conflicting results may also be considered with another point of view. Indeed, one could infer that body modifications may be more an expression of adaptive processes among BPD patients than of severity pathology. For example, getting a tattoo or a piercing may be seen as a way to express rebellion without harming oneself, a process that has been described by Marsha Linehan as a potential distress tolerance skill name “alternate rebellion”. Thus, following this hypothesis, undergoing body modifications may in fact be useful in some ways, particularly for the reduction of the number and/or intensity of emotional crisis. However, this hypothesis is challenged by the previous results we presented, notably on the link between body modifications and NSSI. Regarding the link between BPD symptoms and body modifications, we found that this positive association was mainly driven by an association with item 5 (“suicide and auto-mutilation”) and item 7 (“chronic feeling of emptiness”). The link with the fifth item is coherent with the previous results we presented and offers another argument supporting the link between body modifications and NSSI. On the other side, the link with the seventh item is less clear and can be interpreted in several ways. Chronic feeling of emptiness has been the subject of a recent review [46], which underlines the difficulty to find a clear definition of this symptom, but found a common theme across the different theoretical frameworks, which is “the disconnection from the self and from other people”. More precisely, if we focus on biosocial models, emptiness can be conceptualized as an attempt to decrease emotional experience, resulting in a dysregulation of personal identity [47]. Thus, behind the construct of emptiness, there seems to be a part of emotion regulation and a part of identity. Regarding body modifications, this can be compared with the association we found between body modifications and emotion regulation, and with a recent study which found that body modifications were associated with BPD features *and* with identity problems, and that identity problems may partially explain the relationship between BPD features and body modification [27]. Thus, there seems to be a close three-way relationship between emotion regulation, identity and body modifications, even if the lack of association with the item 3 of the SCID “Identity disturbance” makes it impossible to conclude on the clear nature of this relationship. Further studies are needed to disentangle and precise this complex relationship.

This study has several limits that should be considered. First, we did not use a standardized scale to assess the presence/number of NSSI. Instead, we designed a new scale and calculated sub-scores based on our clinical experience, which limits the interpretation of the data we extracted using this tool. Second, almost 4 out of 5 patients were female, which limits the meaning of our data for male BPD patients, which are known to be as numerous as female patients in the general population [48]. Third, our mean age sample was 29.64, which is quite young but also not surprising given the known amelioration of BPD symptoms over the years [49]. However, given the association between age and body modifications score we found (with younger subjects having more piercings and higher score on the body modifications total score), our results may be less valid regarding the older BPD patients that we also may encounter in our daily practice. Finally, the study’s transversal design and the nature of statistical associations make it impossible to draw firm conclusions on the causal role played by each member of an association.

However, we believe that our results raise the potential interest of body modifications assessment in BPD. On a clinical level, many dimensions of BMs can be investigated when facing a patient with BPD. The number, motives and functions of BMs should be assessed and potentially added to psychoeducation, with patients being taught the potential link between body modifications, emotion regulation, NSSI, and chronic feelings of emptiness. This could be relevant especially in patients undergoing numerous BMs in a period of crisis, to try to distinguish *symptomatic* BMs (secondary to borderline symptomatology) from *common* BMs (with motives similar to the general population) and diminish the potential consequences and regrets after the improvement of BPD symptoms in the case of symptomatic BMs. On a research level, further studies should consider the meaning of different anatomic locations, because one could infer that – for example—earlobe piercings have potentially different motives than genital piercings. It could also be of great interest to consider the potential interpersonal meanings of BMs, given its central aspects in BPD, for example the use of BMs to take part in a specific social group or the use of BMs to cope with difficulties in emotional *expression*.

## Conclusions

This study provides evidence on the prevalence of BMs in BPD patients, and on the link between BMs and NSSI in this population, suggesting a role of emotion regulation in the link between both constructs. These results also suggests that tattoos and piercings may be differentially linked to specific underlying psychological mechanisms. This calls for further consideration of body modifications in the assessment and care of BPD patients.

## Supplementary Information

**Additional file 1.** The Suicidal Behaviors and Body Damages & Modifications Scale. 

**Additional file 2.** Description of non-significant associations.

## Data Availability

The datasets used and/or analyzed during the current study are available from the corresponding author on reasonable request.

## Abbreviations

ADHD: Attention Deficit Hyperactive Disorder
BMs: Body modifications
BMtot: Total score of body modifications
BPD: Borderline personality disorder
BDI: Beck Depression Inventory
BSL-23: Borderline Symptom List – 23 items
CERQ: Cognitive Emotion Regulation Questionnaire
DERS: Difficulties in Emotion Regulation Scale
NSSI: Non-suicidal self-injury
PercTot: Total number of piercing score
SBBDM-S: Suicide Behaviors and Body Damage & Modifications Scale
SCID-BDL: Structured Clinical Interview for DSM-V Personality Disorders5
STAI: State Trait Anxiety Inventory
TatTot: Tattoo-covered body surface percentage score
UPPS-P: UPPS-P Impulsive Behavior Scale
