# Supplementary material for: Body modifications in borderline personality disorder patients: prevalence rates, link with non-suicidal self-injury, and related psychopathology
Source: Borderline Personal Disord Emot Dysregul. 2023 Mar 2;10:7. doi: 10.1186/s40479-023-00213-4 (PMC9979398; doi:10.1186/s40479-023-00213-4)
Supplement: Supplementary file 1 — Additional file 1. The Suicidal Behaviors and Body Damages & Modifications Scale. [file 40479_2023_213_MOESM1_ESM.docx]

**Additional File 1. The Suicidal Behaviors and Body Damages & Modifications Scale**

This questionnaire focuses on different behaviors that you may have engaged in during your life. It is divided into three parts: suicidal behavior, cosmetic body modification, and self-damaging behavior. Please read each question carefully and answer as honestly as possible.

**A. Suicidal Behaviors**

1. Have you ever tried to end your life?
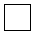
 NO
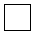
 YES

2. If YES: How many times have you tried to do so? ………..

3. How old were you the 1st time you tried to end your life? ……..

4. How did you try to end your life? Check the method used in the most serious attempt (only one box possible):

*1. Medication
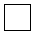

2. Drugs (e.g. alcohol) / Substances (e.g. detergent)
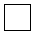

3. Veinosection / Cutting type of bodily injury
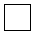

4. Voluntary road accident
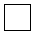

5. Jump from height
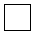

6. Hanging
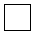

7. Drowning
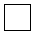

8. Strangulation
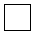

9. Immolation
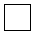

10. Firearm
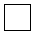
*

*11. Other: ..........................................*

**B. Body modifications for aesthetic purposes**

5. Have you ever had one or more tattoos done by a professional?
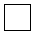
 NO
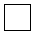
 YES

6. If YES, what percentage of your body is covered with tattoos?


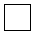
 Less than 10%
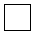
 between 10% and 50%
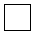
 between 50% and 90%
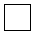
 more than 90%

7. Have you ever had one or more piercings done by a professional?
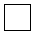
 NO
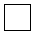
 YES

8. If YES, how many piercings do you have? ……….

**C. Self-damaging behaviors (harming body integrity, aiming to reduce inner distress, with no real intention to die)**

9. Have you ever intentionally harmed yourself?
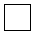
 NO
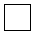
 YES

10. If YES, how often have you engaged in this type of behavior?

|  | 1 time | 1 to 5 time | 5 to 10 times | > 10 times |
| --- | --- | --- | --- | --- |
| 1. Have you ever intentionally prevented a wound from healing? |  |  |  |  |
| 2. Have you ever scratched yourself to the point of bleeding? |  |  |  |  |
| 3. Have you ever punched yourself in the face? |  |  |  |  |
| 4. Have you ever hit your head or thrown your fists or feet against a wall or against a wall or other solid surface? |  |  |  |  |
| 5. Have you ever pulled out your hair or eyebrows? |  |  |  |  |
| 6. Have you ever bitten yourself so hard that you left a mark on your skin? |  |  |  |  |
| 7. Have you ever scarred your arm, legs or any other part of your body? |  |  |  |  |
| 8. Have you ever burned yourself with a cigarette, lighter or any other any other incandescent object? |  |  |  |  |
| 9. Have you ever stuck objects in your body (needles, pins, staples, etc.)? |  |  |  |  |
