# Supplementary material for: Body modifications in borderline personality disorder patients: prevalence rates, link with non-suicidal self-injury, and related psychopathology
Source: Borderline Personal Disord Emot Dysregul. 2023 Mar 2;10:7. doi: 10.1186/s40479-023-00213-4 (PMC9979398; doi:10.1186/s40479-023-00213-4)
Supplement: Supplementary file 2 — Additional file 2. Description of non-significant associations. [file 40479_2023_213_MOESM2_ESM.docx]

**Additional File 2. Description of non-significant associations**

*Associations with* *the total number of body modifications score (BMTot score)*

We did not find significant associations between the BMTot score and the BSL-23 mean score (b=0.09; p=0.611; 95%CI from -0.27 to 0.47), an history of suicide attempt (b=-0.019; p=0.955; 95%CI from -0.70 to 0.66), the BDI total score (b=0.15; p=0.366; 95%CI from -0.17 to 0.49), the STAI-trait score (b=0.07; p=0.656; 95%CI from -0.25 to 0.39), the CERQ adaptive and non-adaptive subscores (b=-0.11; p=0.501; 95%CI from -0.44 to 0.21 and b=0.25; p=0.147; 95%CI from -0.08 to 0.58), or any of the UPPS subscores.

*Associations with the total number of piercings score (PercTot score)*

We did not find significant associations between the PercTot and the BSL-23 (b=0.11; p=0.539; 95%CI from -0.25 to 0.47), an history of suicide attempts (b=0.13; p=0.727; 95%CI from -0.59 to 0.85), the BDI total score (b=0.01; p=0.357; 95%CI from -0.02 to 0.04), the STAI trait score (b=0.07; p= 0.689; 95%CI from -0.26 to 0.41), the CERQ adaptive or non-adaptive subscores (b=-0.08; p=0.653; 95%CI from -0.89 to 0.26 and b=0.24; p=0.166; 95%CI from -0.10 to 0.59), or any of the UPPS subscores.

*Associations with* *the tattoo-covered body surface percentage score (TatTot score)*

We did not find significant associations between the TatTot score and age (b=0.03; p=0.847; 95%CI from -0.30 to 0.37), the SCID total score (b=0.27; p=0.127; 95%CI to -0.08 to 0.61), the BSL-23 mean score (b=0.06; p=0.696; 95%CI from -0.27 to 0.41), NSSIs (b=0.46; p=0.023; 95%CI from 0.06 to 0.87), an history of suicide attempts (b=0.18; p=0.621; 95%CI from -0.53 to 0.89), the BDI total score (b=0.19; p=0.268; 95%CI from -0.15 to 0.54), the STAI-trait score (b=0.05; p=0.763; 95%CI from -0.29 to 0.39), the CERQ adaptive or nonadaptive subscores (b=0.03; p=0.849; 95%CI from -0.33 to 0.41 and b=-0.07; p=0.708; 95%CI from -042 to 0.28) and the “lack of perseverance” and “negative urgency” UPPS subscores.
